# Developmental changes in visual short-term memory in infancy: evidence from eye-tracking

**DOI:** 10.3389/fpsyg.2013.00697

**Published:** 2013-10-02

**Authors:** Lisa M. Oakes, Heidi A. Baumgartner, Frederick S. Barrett, Ian M. Messenger, Steven J. Luck

**Affiliations:** Department of Psychology, Center for Mind and Brain, University of California, DavisDavis, CA, USA

**Keywords:** visual short-term memory, eye-tracking, infancy

## Abstract

We assessed visual short-term memory (VSTM) for color in 6- and 8-month-old infants (*n* = 76) using a one-shot change detection task. In this task, a sample array of two colored squares was visible for 517 ms, followed by a 317-ms retention period and then a 3000-ms test array consisting of one unchanged item and one item in a new color. We tracked gaze at 60 Hz while infants looked at the changed and unchanged items during test. When the two sample items were different colors (Experiment 1), 8-month-old infants exhibited a preference for the changed item, indicating memory for the colors, but 6-month-olds exhibited no evidence of memory. When the two sample items were the same color and did not need to be encoded as separate objects (Experiment 2), 6-month-old infants demonstrated memory. These results show that infants can encode information in VSTM in a single, brief exposure that simulates the timing of a single fixation period in natural scene viewing, and they reveal rapid developmental changes between 6 and 8 months in the ability to store individuated items in VSTM.

## Introduction

Visual short-term memory (VSTM) is critically important for normal processing of the visual world. Adults use it thousands of times each day to perceive continuity across eye blinks and eye movements (Irwin, 1991; Hollingworth and Henderson, 2002), and to compare visual objects that cannot be simultaneously foveated (Pomplun et al., 2001). In this memory system, representations are rapidly formed in an all-or-none manner (50 ms per object: Vogel et al., 2006; Zhang and Luck, 2009), capacity is extremely limited, and the information is maintained only briefly, until it is replaced with new information. VSTM is clearly a *working memory* (WM) system, because it is used in the service of other processes (for a review, see Luck, 2008).

VSTM must emerge in infancy. Young infants plan, execute, and correct eye movements (Aslin and Salapatek, 1975; Hainline et al., 1984; Richards, 2001), and in the first year they integrate information encountered before and after episodes of occlusion (Arterberry, 1993; Spelke et al., 1995). The challenge is how to assess this memory system in infants. To isolate VSTM, research with adult participants has used change detection tasks with very brief encoding and retention periods (e.g., several 100 ms) (Luck and Vogel, 1997). In this *one-shot change detection* task, memory is assessed after a single stimulus cycle (a single “shot”). Trials have the following sequence: a *sample array* (e.g., a set of colored squares) is briefly presented, this is followed by a short retention interval (usually 1000 ms or less), and then finally a *test array* that is either identical to the sample array or contains one difference (e.g., one item that has changed color) is presented. Participants indicate whether or not the test array is the same or different from the sample array (Vogel et al., 2001; Alvarez and Cavanagh, 2004; Todd and Marois, 2004; Cowan et al., 2005) or report the location of the changed item (Gold et al., 2006; Hyun et al., 2009; Johnson et al., 2013). The timing is designed to reflect natural vision, in which a brief period of fixation is followed by a short gap (reflecting the suppression of vision during a saccade or eyeblink) and then another period of fixation.

**Figure 1:**
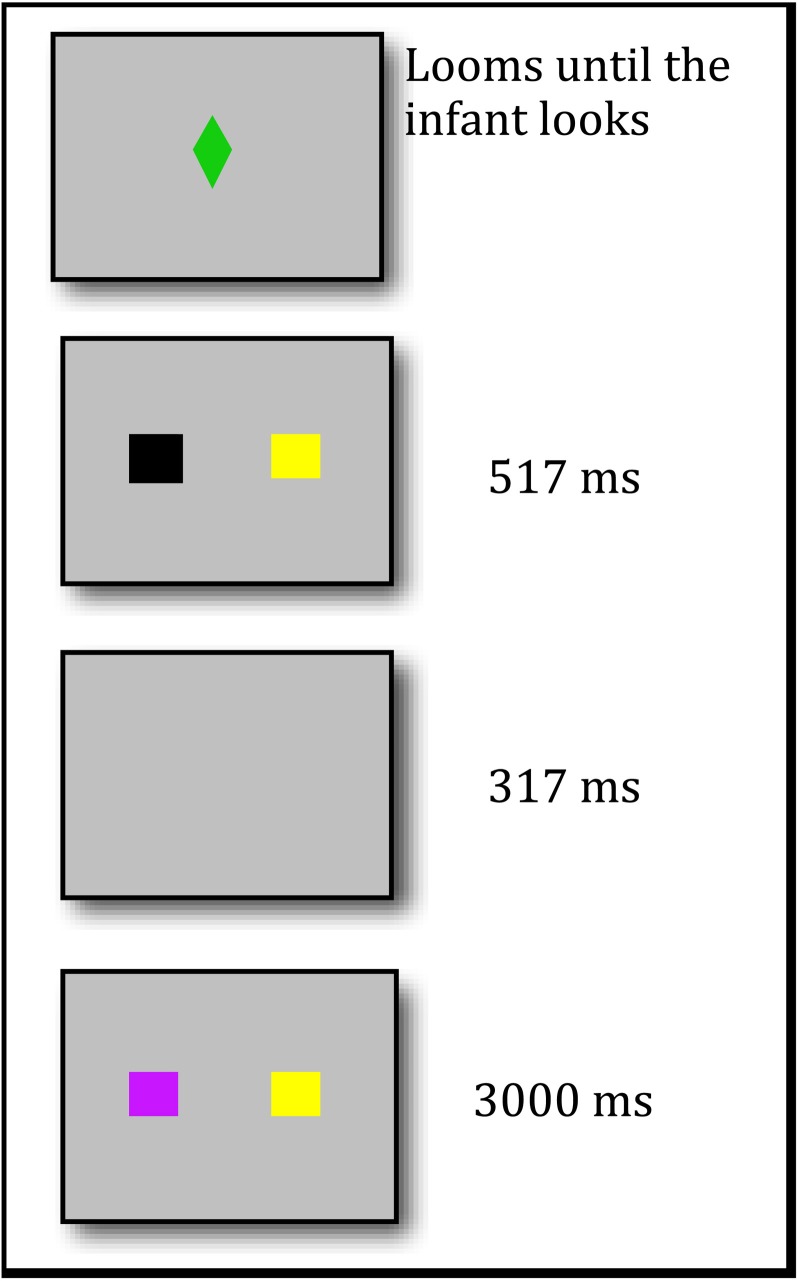
**A Sequence of events in a one-shot change detection trial**.

Although infants' memory has been examined using study and retention periods of tens of seconds (or less) (Fagan, 1990), and procedures have been developed to minimize the use of *long-term memory* (LTM) in infants' responding (Ross-Sheehy et al., 2003), it is extremely difficult to design a task that isolates VSTM in infancy in the same way that this system has been isolated in adults. Therefore, we developed an infant version of the change-detection task using eye-tracking (see Figure 1). Our *one-shot change detection task* has temporal features like those used in adult change detection tasks, and includes trials with the following sequence: a sample array consisting of two colored squares is briefly presented (for 517 ms), followed by a short retention interval (317 ms), and finally a test array in which one of the two squares has changed color is presented for 3000 ms. Thus, this task uses extremely brief exposure and retention periods. Moreover, given these timing parameters, the presentation of the test array will overwrite any iconic memory for the sample array, thus eliminating the possibility that change detection is a function of iconic memory (Becker et al., 2000). In addition, item colors are drawn from a small set of possible colors (which will cause interference with LTM). Therefore, if infants fixate the changed color more than the unchanged color, then they must have formed a VSTM representation of at least one item from the sample array.

This task has much in common with the *simultaneous streams task* we previously developed to assess VSTM in infancy (Ross-Sheehy et al., 2003). This previous task used a similar timeframe for encoding and retention: arrays of items (e.g., colored squares) repeatedly appeared briefly (e.g., for 500 ms) and disappeared briefly (e.g., for 300 ms). We presented on a series of trials, two stimulus streams side-by-side, each that involves an array of items that repeatedly appears and disappears continuously for a period of several seconds. One stream on each trial is a *changing* stream (e.g., the color of a randomly selected item changes each time the array reappears). Given the temporal features of the cycling, *detecting* that the array changed from onset-to-onset requires that infants rapidly encode each array (or a subset of the items in each array) into VSTM. The other stream presented on each trial is a *non-changing* stream (e.g., all the items remain the same from cycle to cycle). If infants prefer the changing stream, the conclusion is that they have stored some information from the streams into VSTM (see Perone et al., 2011).

Using this simultaneous stream task, we have observed significant preferences for the changing stream in infants as young as 4 and 6 months of age, at least when each stream contained only a single object (Ross-Sheehy et al., 2003). Although this probably reflects the use of VSTM to store items and detect changes from one cycle to the next, a preference for the changing stream may also reflect a gradual habituation to the non-changing stream, which contains the same color on each cycle for many consecutive cycles (note that recognizing that the item did not change color would require encoding information in VSTM). To rule out this possibility, it is necessary to use a procedure in which the non-changing information is presented only once. Therefore, the one-shot procedure used in the present study can provide definitive evidence of VSTM.

Our one-shot task also has much in common with procedures developed to assess aspects of WM in infancy. In such procedures, infants must store visual information temporarily, and success over trials requires discarding stored information (Feigenson and Carey, 2003; Káldy and Leslie, 2003, 2005; Kibbe and Leslie, 2011; Káldy and Blaser, in press). For example, Káldy and Blaser (in press) presented 6-month-old infants two items for 4 s, one to the right and one to the left of midline. The items were occluded for 2.75 s and then unoccluded to reveal that one item has changed (e.g., a green item has been changed to red). On each trial, infants made more of their first fixations to the item that changed color than to the unchanged item. Importantly, the location of the changed item varied from trial to trial, so infants' systematic preference for the change cannot reflect LTM representations of the location of the red item over trials. However, because the encoding and retention periods are several seconds on each trial, it is not clear that such tasks tap precisely the same memory system as does the change detection task used with adults.

Using our one-shot change detection task, we sought to provide additional understanding of rapid changes in VSTM between 6 and 8 months. Studies using the simultaneous streams task have consistently reported developmental change in infants' memory for items in multiple item arrays between 6 and 8 months of age. Infants 6 months and younger fail to prefer a change when the identities of individual items change in multiple item arrays (e.g., arrays with 2 or 3 items, Ross-Sheehy et al., 2003), even when every item in the array changes (Oakes et al., 2006, 2009). Infants at 8–10 months, in contrast, prefer changing streams over non-changing streams even when each stream contains multiple items (Ross-Sheehy et al., 2003; Oakes et al., 2006, 2009, 2011).

The question is *why* do infants 6 months and younger fail to prefer changes in multiple-item arrays and older infants succeed? One possibility is that infants' actual VSTM *capacity* develops; that is, younger infants can only encode in VSTM one item and older infants can encode in VSTM multiple items. In support of this possibility, Káldy and Leslie found in a very different WM task that when 6-month-old infants observed two objects hidden sequentially behind an occluder, they remembered the last object hidden but not the first object (Káldy and Leslie, 2005) 9-month-old infants apparently remembered both hidden items (Káldy and Leslie, 2003).

The evidence from the simultaneous streams task is not that clear. In this task we do not assess infants' response to the individual items that changed, but rather we assess their response to the array as a whole. Logically, if young infants respond to a change at set size 1 but not set size 3, it is possible that they did not prefer the change at the large set size because they were remembering only one of the items on each cycle—sometimes it was the item that changed and sometimes it was not. However, the overall pattern of results is inconsistent with this explanation. First, young infants failed to prefer changing streams *even when every item changed on each cycle* (Oakes et al., 2006, 2009). Detecting a change in such arrays should be trivial even if one encodes the information from only one of the items in the array. Second, when faced with multiple-item arrays, young infants seem to process more global properties, detecting changes in features such as the number (Libertus and Brannon, 2010) and configuration (Oakes et al., 2011). Thus, the overall pattern of results suggests that the change in capacity reflects a different mechanism: when presented with multiple-item arrays, older infants can represent the features of the individual items but younger infants cannot, representing instead more global properties of the arrays. We propose that the capacity changes uncovered by the simultaneous streams task may be the result of developmental changes in the ability to rapidly *individuate* multiple items presented simultaneously, attend to those individual items, and encode the features of those items. Indeed, the process of *individuating* items, or using the spatial locations of items to form initial, coarse representations of those items (enough detail to select the items from the background, but not enough detail for object identification), may be central to VSTM. Xu and Chun (2009) argued that a first step to encoding and perceiving the items in a crowded visual scene is to individuate them.

Infants' responding in our one-shot task can begin to address this possibility. If infants individuate the items in multiple item arrays, attend to those items, encode the features of the items into VSTM, and detect when an individual item changes, then they will look longer at the changed *item* in the one-shot task. This is precisely what Káldy and Blaser (in press) observed in 6-month-old infants who were given 4 s to learn the identities (and locations) of the two items. The relatively long study period may have allowed them to individuate two simultaneously presented items. We asked here whether infants also can individuate two items in an array rapidly, using a timecourse that more closely resembles a single period of fixation in natural vision.

We assessed infants' responding in our task using high-resolution eye-tracking. Eye-tracking is becoming common in studies of cognitive development, but these studies rarely take advantage of the high temporal resolution afforded by eye-trackers, which typically record eye position 30 or more times per second. One reason for this is that the large number of individual data points creates a problem of multiple statistical comparisons. We therefore, developed a novel and powerful data analysis strategy—based on permutation statistics that have become popular in neuroimaging and electrophysiological studies (Groppe et al., 2011; Maris, 2012)—that allows the time course of a difference between conditions to be precisely determined while controlling for multiple comparisons. This strategy could easily be used by researchers who are using eye-tracking to study a variety of developmental issues.

We conducted 2 experiments. In Experiment 1, we assessed 6- and 8-month-old infants' sensitivity to a change in the color of one item in the one-shot task when the initial sample array contained two different colored items. In Experiment 2, we asked whether 6-month-old infants' performance was improved when the initial sample array contained two identical items, which reduced the need to individuate the items.

## Experiment 1

### Method

#### Participants

The final sample consisted of 26 6-month-old infants (*M* = 193.15 days, *SD* = 8.49; 13 boys) and 26 8-month-old infants (*M* = 245.73 days, *SD* = 9.19; 15 boys). All infants in this and the following experiment were healthy and typically developing with no known vision problems. To reduce the likelihood of including colorblind infants in our sample, we excluded infants whose familial history put them at significant risk of colorblindness (male infants with maternal familial colorblindness, and female infants with maternal familial colorblindness and whose father was colorblind). The infants were of mixed ethnic and racial backgrounds: 36 infants were White, 3 infants were Asian, 1 infant was Native American, 10 infants were mixed race, and race was not reported for 2 infants. Regardless of race, 10 infants were Hispanic. The 51 mothers who reported education level had graduated high school, and 46 mothers had completed at least a Bachelors degree.

Infant names were obtained from the state Department of Public Health. Parents were sent informational packets and contacted us by phone, e-mail, or return post-card to indicate their interest in participating. When their infant approached the appropriate age, parents were contacted by phone or e-mail to set up an appointment if they wished. Parents were not compensated, but their travel expenses were reimbursed upon request, and all infants were given a certificate and a small toy, t-shirt, or bib in appreciation of their participation.

An additional 12 infants were tested but not included in the final analyses due to fussiness or failure to contribute more than 6 trials of useable data (*n* = 9), inability to calibrate (*n* = 1), or equipment error (*n* = 2).

#### Apparatus

We collected eye-movement data using an Applied Science Laboratory (ASL) pan/tilt R6 eye-tracker fitted with a magnetic head tracker (Ascension Flock of Birds) and controlled by a Dell computer. The eye camera was located below and in front of a 37' Westinghouse LCD monitor (16:9 aspect ratio) on which the stimuli were presented. The ASL eye camera was focused on the infant's right eye; a small wide-angle camera connected to the eye camera allowed the experimenter to monitor the infant's behavior in general.

Point-of-gaze (POG) was determined using the recorded pupil and corneal reflection from an infrared light source. If the infant's eye fell out of range of the eye camera, the system used information from the Ascension head tracking system about the infants' head position (via a sensor embedded in an infant-sized headband, positioned just above the infant's right eye) to quickly adjust the camera.

A second Dell computer was used to present stimuli on the Westinghouse monitor, and to send signals to the eye-tracking computer that were stored with gaze data indicating when particular events occurred (e.g., the start of the trial). These codes allowed us to coordinate the infant's eye movements with the stimuli.

#### Stimuli

Each experimental trial began with a single sample array consisting of two 11 × 11° colored squares, each centered 9° from the center of the monitor (the bottom of the squares was 9.75° from the bottom of the screen) (these values assume a 100-cm distance from the screen). The two colors were selected at random from a set of 8 colors with the following CIE [x, y, luminance] coordinates: red [0.63, 0.34, 28.7], green [0.26, 0.54, 25.4], brown [0.44, 0.43, 28.9], yellow [0.38, 0.48, 209], orange [0.47, 0.43, 89.9], black [0.28, 0.34, 0.9], cyan [0.22, 0.33, 94.1], and violet [0.22, 0.13, 27.6]. After ~517 ms, the two squares disappeared, and the screen was blank for ~317 ms. Two squares then reappeared in the same locations, but now one of the colors of the squares had changed to one of the remaining colors. This array remained visible for 3000 ms (see Figure 1).

Color is a three-dimensional feature with dimensions of hue, saturation, and luminance, and the eight colors in this experiment varied along all three dimensions to maximize the differences among color values and therefore minimize any effects of imprecision in feature coding. The colors used on a given trial were selected at random, so the changed item was equally likely to be clockwise or counterclockwise in hue, greater or less in saturation, and greater or less in luminance relative to the unchanged item. Therefore, any systematic preferences for the changed item cannot be attributed to a preference for a particular range of stimulus values (e.g., a preference for brighter stimuli).

We used several other stimuli in this experiment to attract the infant's attention to the monitor. At the start of each trial, we presented at the center of the screen a sequence of shapes, each selected at random from a large set of shapes (diamond, star, etc.), that loomed from 0 × 0° to 16 × 16° (at 100 cm viewing distance) in ~800 ms, accompanied by a randomly selected sound (e.g., boing, ring, clunk), disappeared, and then was replaced by another randomly selected shape. During periods of inattention or fussiness, we presented one or more of the following stimuli: (1) a series of photos of babies' faces accompanied by children's music, (2) animated animals singing a lively song, (3) brief clips from a variety of children's television shows, including *Blues Clues*, *Teletubbies*, and *Sesame Street*, and (4) a collection of shapes that moved in random ways across the monitor accompanied by a beeping sounds.

#### Procedure

All procedures were reviewed by the Institutional Review Board of the University of California at Davis. Two highly-trained experimenters conducted the session: an eye-tracking experimenter who controlled the eye-tracking system and a stimulus-presentation experimenter who controlled stimulus presentation.

The infant sat on a parent's lap ~100 cm from the video monitor and 75 cm from the eye camera. Parents were positioned on a fixed-position chair without wheels and could not move the chair forward or back. The infant could lean forward and back so that his or her distance from the video monitor could range between 95 and 115 cm and remain in focus of the eye-tracker. A curtained wall hid the equipment and experimenters. Once the eye-tracker was focused on the infants' eye, we initiated a 2-point calibration scheme to calibrate the infant's POG to the eye-tracker. Looming circles were presented at calibration points ~8.2° above and 8.2° to the left of the center of the screen and ~8.2° below and 8.2° to the right of the center of the screen. The eye-tracking experimenter pressed a key when he or she judged that the infant looked at the onset of the calibration stimulus. To check calibration, the looming circle was presented at other calibration points (e.g., 8.2° directly to the left, 8.2° to the left and 8.2° below, and 8.2° directly to the right of the center of the screen), and remained visible until the cross-hairs clearly indicated that the infant looked at that item. The position of the cross-hairs on the looming circle provided a visual verification of the calibration. When the infant moved his or her eyes to the new target, cross-hairs superimposed on an image of the stimulus would indicate where the infant was looking. If the calibration was good, the cross-hairs were positioned on the circles; if the calibration was poor, the cross-hairs would be systematically off the circles (e.g., above the circles, to the left), and the calibration procedure was reinitiated. This procedure typically took less than 2 min and was quite effective (we eliminated only 1 infant due to calibration failure).

The main experiment involved a series of one-shot change detection trials. Before each trial, the attention-getter was presented in the center of the monitor. The attention-getter was presented until the stimulus-presentation experimenter judged (on the basis of the cross-hairs) that the infant was fixating on the attention-getter. The experimenter then pressed a key to initiate the trial. We used this interactive system to allow us to flexibly respond to each infants' style and attention level and thereby maximize the number of trials they contributed to the data analyses.

For each trial, the experimental script randomly selected the colors of the two squares for the sample array, determined whether the changing stimulus would appear on the left or right, and randomly selected the color for the changed square from the remaining six colors. Each infant received a different order of stimuli, randomly determined given the constraints of the experimental design. To help maintain the infant's interest level, each trial was accompanied by a randomly chosen classical music selection (clips of pieces by Bach, Mozart, Pachelbel, and Ravel); although not always explicitly stated, the use of music to maintain interest and attention is common in infant eye-tracking procedures.

The stimulus-presentation experimenter could interactively present the reorienting stimuli between trials if the infant became inattentive, or if the eye-tracker lost focus on the eye. The experimental session continued until the infant became fussy or stopped looking at the monitor, or until 64 trials had been completed.

#### Data processing

Infants' POG was recorded at a rate of 60 Hz. To minimize noise in the fixation data, each recorded data sample represented a running average of 4 online samples (i.e., the current sample and the 3 previous samples). As a result, there was a delay of ~1.5 samples (or 25 ms) between when we recorded the gaze data and the best estimate of when that (average) gaze occurred. At each sample, the horizontal and vertical coordinates of this averaged eye position were recorded along with codes sent by the stimulus presentation software. Our stimulus presentation program, created in Adobe Director, sent codes via the computer's parallel port directly to the eye-tracker control unit indicating the onset of the sample array, the onset of the test array (and whether the change was on the left or right), and the end of the trial. Using a dual channel oscilloscope and a photovoltaic light sensor, we determined that the codes were recorded ~33 ms before the stimulus appeared. In addition, there was a small and variable delay of ±8.33 ms depending on when during the 16.67 ms sample period the signal was received. We therefore, adjusted the timing of stimulus onset for our analyses, taking into consideration all of these sources of slight temporal variation in the synchronization between stimulus onset and the eye tracker signal.

A blink filter was implemented in which pupil loss of <12 samples was considered a blink; when the pupil was lost for 12 or fewer samples the location of the eye was interpolated from the available data (i.e., the position was frozen and that position was used as the eye-position for each of the missing samples). When there were no pupil values for more than 12 samples, no gaze was recorded for that period.

We used custom Matlab routines to evaluate the number of observed samples (and duration of looking) that fell in three rectangular Areas of Interest (AOIs); two AOIs (28° by 16°) were centered on the locations of the two squares, and the third AOI (2° by 6.5°) was centered on the central attention getter. Our AOIs were somewhat larger than the actual squares (which were 11 × 11°) to account for calibration imprecision and for the ability of the participants to use parafoveal vision to perceive color.

Data from a given trial were included only if they met the following two criteria: (1) the infant spent at least 100 ms of the sample period with his or her eyes directed toward the stimulus regions (i.e., in one of the three AOIs)—in other words, we only included trials on which the infant had an opportunity to perceive the sample stimuli, and (2) the infant accumulated at least 200 ms of total looking within the two object AOIs during the test array—this allowed us to include only trials in which infants had at least one clear fixation to one of the two squares. In addition, we excluded any infant who showed a general lack of interest in the task by contributing 6 or fewer trials that met the above criteria (*n* = 9). Looking times for these infants were very unstable; they tended to exhibit extreme side biases, long periods of fussiness, etc.

**Figure 2:**
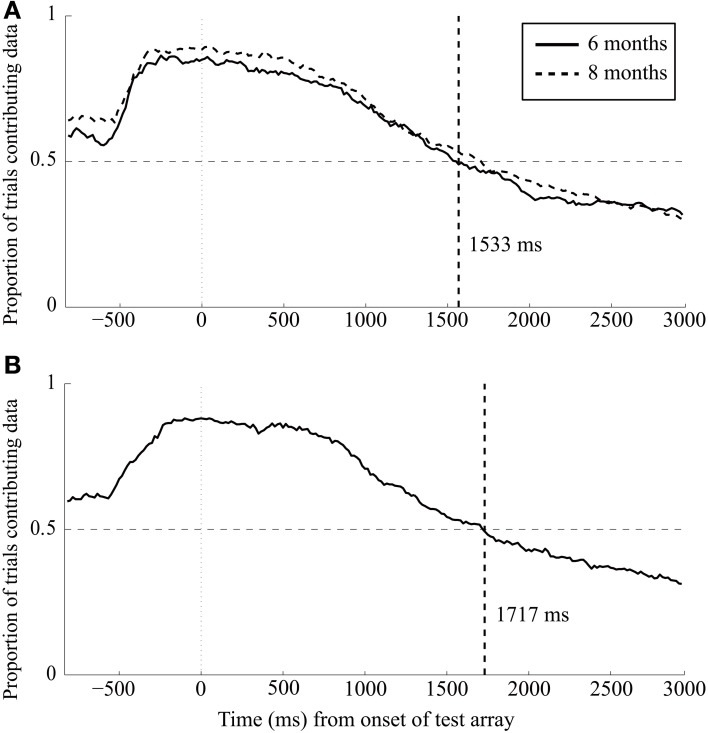
**Proportion of trials with looks to the left or right AOI at each sample for 6- and 8-month-old infants in Experiment 1 (panel A) and 6-month-old infants in Experiment 2 (panel B).** Note that for all samples, the proportion of trials with looks increases dramatically over the pre-change period (from −833 to 0 ms), and then begins to decrease during the post-change period. The dashed vertical line indicates the 50% cut-off used to determine the end of the analysis window used in each experiment.

Inspection of the data indicated that infants rarely looked at the test array for the entire 3000-ms duration of this array. To examine how infant interest in the two target AOIs changed over time, we calculated the proportion of trials for which infants were looking toward the two stimulus locations (the left or right AOIs) at each sample point (aggregated across subjects) for the entire trial. These data are presented in Figure 2A. A value of 1 in this figure indicates that every infant looked toward the stimulus locations at that sample point on every trial and a value of 0 indicates that none of the infants looked at the stimulus locations at that point on any of the trials. A value of 0.5 indicates that gaze was directed toward the stimulus locations at that point for half of the trials across all the infants. The figure is divided into the pre-change period (−833 to 0 ms) and the post-change period (0–3000 ms) (note that because this figure does not include looks to the center AOI, the proportion of trials for which we recorded looking during the sample period is underrepresented, especially at the beginning of the trial). During the pre-change period, gaze rapidly shifted between the two target AOIs and a high proportion of trials include looking to one of those AOIs in the 300 ms before the change occurred, and remained on one of those AOIs when the test array was presented.

The critical period is the post-change period—although gaze was directed toward the two stimulus locations on a relatively high proportion of the samples immediately after the change, the number of trials in which gaze was directed at those locations dropped dramatically over time, and dipped below 50% ~1500 ms after the onset of the test array. To focus our data analyses on the portion of this time period in which most infants were actually looking at the stimuli, we ended our analysis point at the time at which eye position was no longer inside one of the two AOIs on few than 50% of the trials. This 50% point occurred at 1533 ms post-change in the 6-month-old infants; to facilitate comparisons across ages, we used the same analysis period for 8-month-old infants as well (although their 50% point occurred slightly later, at 1700 ms). In addition, our analysis period began 200 ms after test array onset because a minimum of 200 ms is typically required to make a saccade on the basis of the match between a stimulus array and memory (Hyun et al., 2009). Thus, the analyses reported here are for the duration of looks to the right and left AOIs from 200 to 1533 ms after the onset of the test array. Trials with no looking to either AOI during this window were also excluded from the analysis.

### Results

Because the median is less influenced by extreme values, all of our analyses are based on the median score across an individual infant's trials, rather than the mean. Note, however, that our analyses represent *group means* of the individual infants' medians.

#### General characteristics of infants' looking

Six- and 8-month-old infants were equally engaged in this task: (1) they contributed similar numbers of trials that met our inclusion criteria, 6 months *M* = 25.12, *SD* = 12.96; 8 months *M* = 29, *SD* = 14.70; *t*_(50)_ = 1.01, *p* = 0.32, (2) they were equally interested during the sample and retention period (833 ms), 6 months *M* = 717.31 ms, *SD* = 83.16; 8 months *M* = 734.29 ms, *SD* = 84.60; *t*_(50)_ = 0.73, *p* = 0.47, *d* = 0.20, and (3) they looked to the displays for approximately the same amount of time during the analysis window of the test period, 6 months *M* = 884.62 ms, *SD* = 220.54; 8 months *M* = 960.90 ms, *SD* = 213.85; *t*_(50)_ = 1.27, *p* = 0.21, *d* = 0.35. Thus, infants at both ages typically had many hundreds of milliseconds exposure to the sample stimuli, which is sufficient for young infants to perceive and encode color information (Catherwood et al., 1996). Similarly, they were equally interested overall during the test phase. Thus, any age differences in infants' change preferences cannot be due to differences in overall interest.

#### Infants' preference for the changed item during the test period

**Figure 3:**
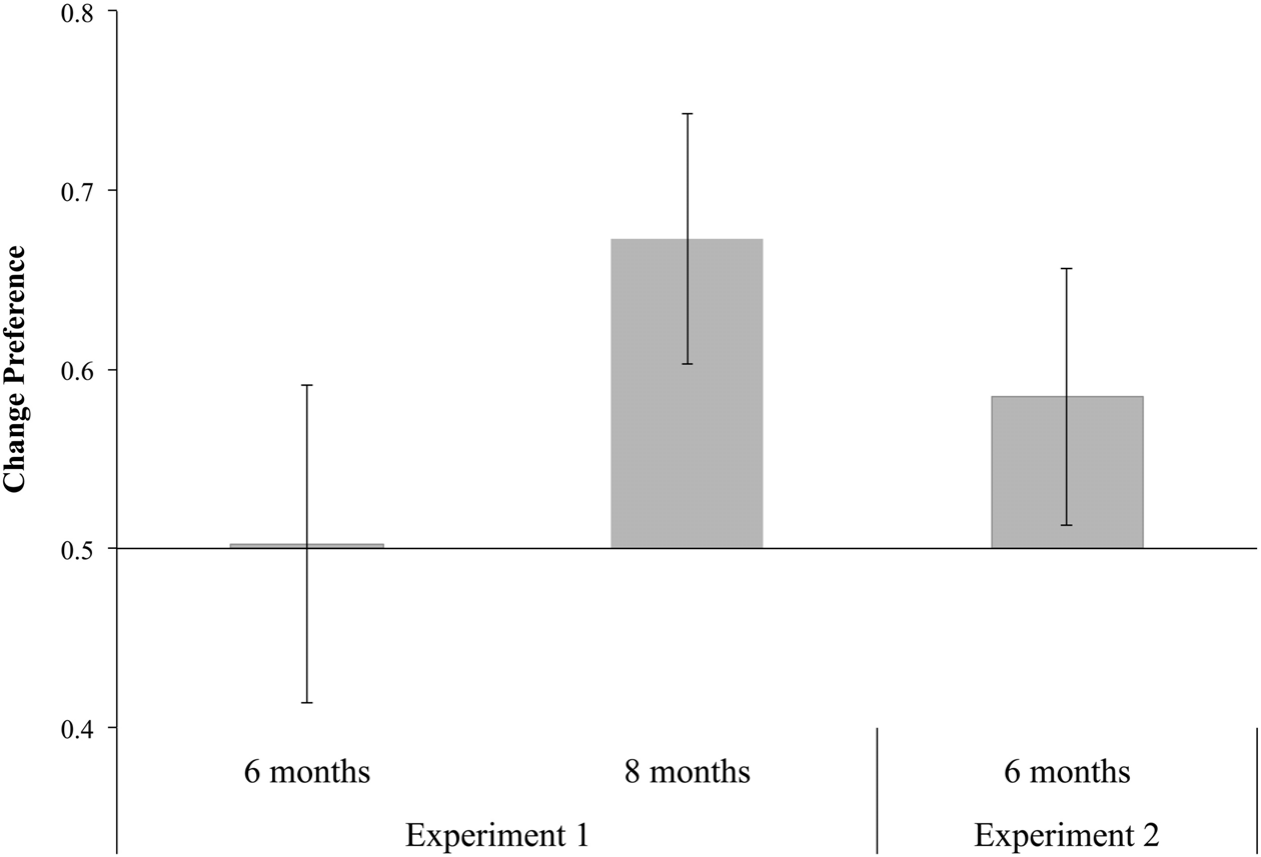
**Average change preference scores for 6- and 8-month-old infants in Experiment 1 and 6-month-old infants in Experiment 2 during the analysis window of the test array (200–1533 ms post-change in Experiment 1, and 200–1717 ms in Experiment 2).** Error bars represent 95% confidence intervals.

To determine how long infants looked at the changed square relative to the unchanged square during the analysis window of the test period, we calculated a *change preference* score by dividing the duration of looking *within* the AOI for the changed item by the duration of their looking to both AOIs combined. If infants prefer the change, this score will be greater than chance, or 0.50. For each infant, we calculated the median change preference score across all trials that met our inclusion criterion, and then entered these median scores into the statistical analyses. The average change preference scores are shown in Figure 3. Eight-month-old infants' change preference scores were significantly greater than chance, *t*_(25)_ = 4.84, *p* < 0.0001, *d* = 0.95, but 6-month-old infants' change preference scores did not differ from chance, *t*_(25)_ = 0.06, *p* = 0.96, *d* = 0.01. Moreover, 8-month-old infants' change preference scores were significantly greater than those of 6-month-old infants, *t*_(50)_ = 2.95, *p* < 0.01, *d* = 0.82.

#### Moment-by-moment gaze behavior toward the changed and unchanged items during test

One advantage of eye-tracking methods is the capability to examine moment-to-moment changes in looking over the course of individual trials to determine the time course of the preference for the changed and unchanged items. Thus, it is possible to determine, for example, whether younger infants exhibited an initial shift of gaze toward the changed item that was followed by a shift of gaze toward the unchanged item, leading to the appearance of no preference when the data were aggregated over time. It also makes it possible to determine whether gaze is immediately attracted to the changed item within 200–300 ms, as in adults (Hyun et al., 2009), or whether the comparison and orienting processes are slower in infants than in adults.

**Figure 4:**
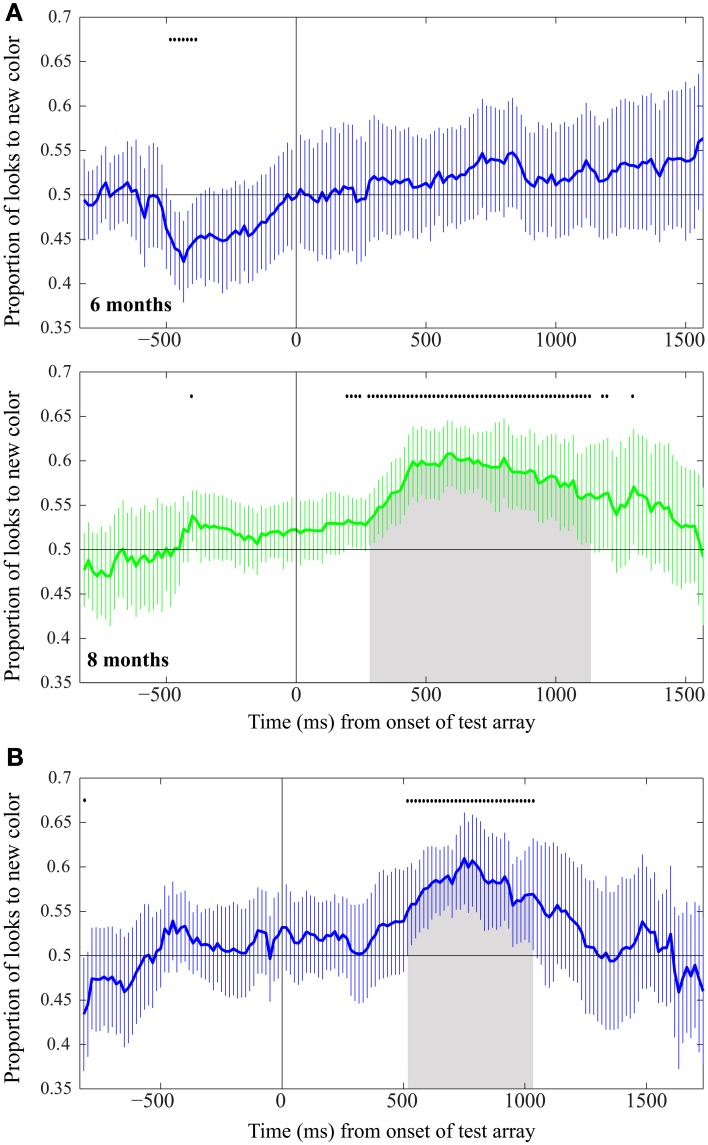
**The subject-weighted proportion of looks to the changed square (1 = 100% of all looks aggregated across subjects were to the changed square; 0 = 0% of all looks were to the changed square) by age and Experiment (Experiment 1 in panel A, Experiment 2 in panel B).** For each observed sample during the sample and delay period (−833 to 0 ms) and during the post-change period (0–1533 ms for Experiment 1 and 0–1717 ms for Experiment 2), the curve represents the mean responding and the vertical lines represent the 95% confidence interval for each sample. The dots above the curve indicate samples that were significantly different than chance (0.50), by an uncorrected two-tailed *t*-test. The shaded area under the curve marks runs of samples that pass the 95% run length criterion.

Figure 4A shows the mean preference for the changed item for each of the 50 samples during the 833-ms pre-change period and the 92 samples during the analysis window for the test display (from 0 to 1533 ms after the onset of the test array; the endpoint of the analysis window determined as described above, and here we include the 200 ms immediately after the onset of the change so we can determine the time course of infants' eye-movements toward the changed item). To compute these values, each sample was coded as 1 (if the infant was looking toward the square that changed color), 0 (if the infant was looking toward the unchanged square), or null (if the infant was not looking to either square). We then averaged across all non-null data points at each sample to calculate the proportion of looking toward the changed item.

Figure 4 shows the average across subjects for each age group during both the pre- and post-change period; the vertical lines represent the 95% confidence interval at each sample. As is illustrated in this figure, the 8-month-old infants exhibited little deviation from chance during the pre-change period, but a strong preference for the changed side beginning ~300 ms after the onset of the test array, and the confidence intervals were beyond chance for many sample periods. Six-month-old infants, in contrast, exhibited a slight preference for the non-change item during the pre-change period (which must have been random variation because they could not have known which item would change) and only a weak preference for the changed item during the post-change period, with confidence intervals that included chance at every sample.

As a first step toward determining the statistical significance of these preferences, we computed a separate *t*-test for each time point (using the preference scores calculated for each subject) to determine whether the mean value at that time point was greater than chance (see Figure 4). During the pre-change period, the 8-month-old infants had one sample that was significantly greater than chance, and the 6-month-old infants had 7 samples that were significantly less than chance (indicated by dots above each curve). No correction for multiple comparisons was applied to these t tests, and because the infants could not know during this period which side would ultimately contain a change, the variations from 0.5 must reflect random variations in eye position. Importantly, none of the samples were different from chance for the time points immediately before the change in either age group; this indicates that there was no bias in eye position at the onset of the test array. After the change, 8-month-old infants had many time points with preference scores significantly greater than chance, whereas 6-month-old infants had no time points with preference scores different from chance.

The use of a large number of individual statistical tests can potentially lead to a large number of false positives. For example, the significant values in the pre-change period must have reflected random variations rather than reliable shifts in gaze toward or away from the location that ultimately contained the change. The Bonferroni correction procedure could be used to address this problem, but this correction would be overly conservative given that the samples are not independent (i.e., eye position at one sample is highly correlated with eye position at nearby samples). A similar problem is faced by EEG/ERP studies (Maris and Oostenveld, 2007; Groppe et al., 2011; Maris, 2012) and neuroimaging studies (Nichols and Hayasaka, 2003). We therefore, adapted a non-parametric analytic approach that has become widely used in these domains to provide a powerful and robust means of determining the time points at which looking was significantly different across groups or conditions (for examples see, Bullmore et al., 1999; Nichols and Holmes, 2001; Hayasaka and Nichols, 2004).

Our approach takes advantage of the fact that a real preference for the changed side should consist of many consecutive samples with the same direction of gaze. To implement this idea, we used the *t*-test results shown in Figure 4 and asked how many consecutive samples were individually significant at the 0.05 level (uncorrected). A consistent preference should lead to a large number of consecutive significant preference scores (a long *run length*), whereas random noise should lead to disconnected bursts of consecutive significant scores (or no significant scores at all). As shown in Figure 4, 8-month-old infants exhibited a long series of consecutive preference scores beginning ~300 ms after the onset of the test array.

The statistical question is whether the 8-month-old infants' run length was greater than would be expected by chance. Because eye position at one time point is strongly predicted by the eye position at the surrounding time points, answering this question is not trivial. Here, we answered this question employing a *resampling* approach that uses random permutations of the actual data to determine the probability that a given run length would occur by chance. In this approach, the codes that indicated which side contained the change were randomly shuffled (permuted) for each trial, and then the data were analyzed just as was done with the original data. If the null hypothesis is true, then it should not matter which side contained the change, and the longest run of consecutive significant points in the permuted data should be similar to the run length observed in the actual, non-permuted data.

Of course, a single random permutation of the codes might lead to a non-representative result. We therefore, conducted 1000 iterations of this procedure, with a different random permutation of the codes on each iteration, and we recorded the maximum run length for each iteration. Each iteration involved the following steps, which were performed separately for each age group: (a) randomly permuting the codes on each trial and computing a change preference for each subject at each sample point; (b) computing *t*-values comparing the mean preference score to chance at each sample point; (c) determining the length of the longest run of consecutive significant sample points (with a value of zero if no samples were significant). The result of each iteration was a single value representing the maximum run length for that iteration, and the probability distribution of these maximum run lengths provides an excellent estimate of the null distribution (the distribution of run lengths that would be expected if the null hypothesis were true). If the maximum run length observed in the actual, non-permuted data is in the top 5% of this null distribution, then this run is considered statistically significant (just as an *F*-value in the top 5% of the *F* distribution is considered statistically significant).

**Figure 5:**
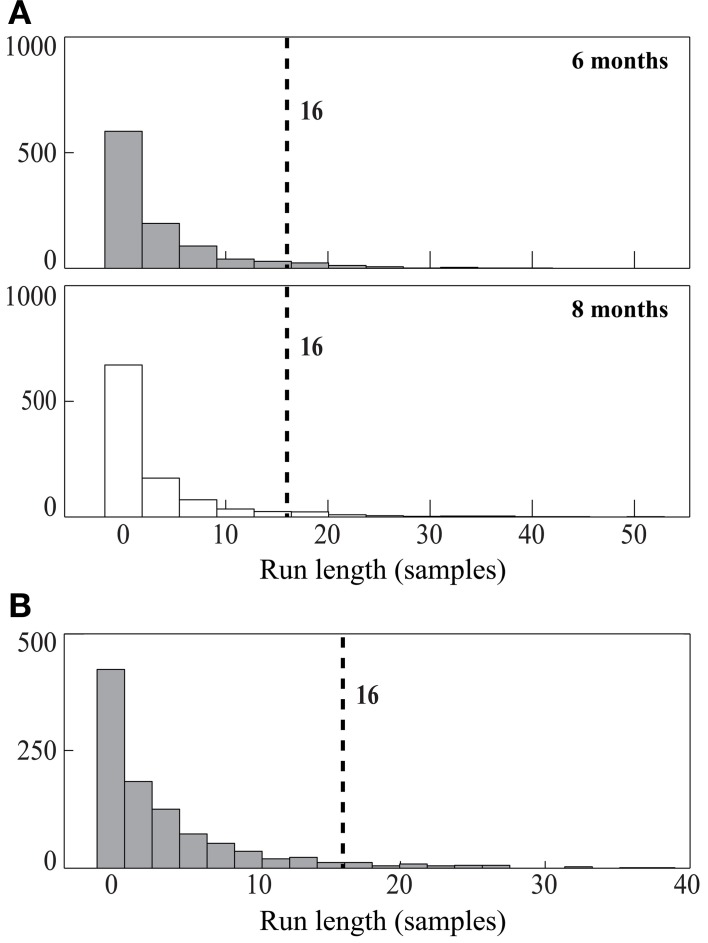
**Null distributions of maximal *run length* from each iteration of the permutation analyses for 6-month-old (gray bars) and 8-month-old (white bars) infants in Experiment 1 (A) and for 6-month-old infants in Experiment 2 (B).** The 95% cutoff value is indicated by the number to the right of the dashed vertical line.

Figure 5A shows the null distributions from this permutation analysis for the 6- and 8-month-old infants. The most common value was zero (no significant samples), and the probability became progressively smaller for longer and longer run lengths. To be considered significantly greater than chance, a run of individually significant preference scores would need to be at least 16 samples long for both 6-month-old and 8-month-old infants (these are the 95% points in the null distributions). When applying these criteria to our observed data, it is seen that whereas the 6-month-old infants had no runs that met these criteria, the 8-month-old infants had a run of 52 consecutive significant preference scores that began 300 ms after the onset of the test array and lasted for 867 ms. This run was substantially longer than would be expected by chance (i.e., it greatly exceeded the 95% point in the null distribution), and it is therefore statistically significant. This converges with the results of the prior analyses, and it pinpoints the time range during which the 8-month-olds exhibited a consistent preference for the changed side (300–1167 ms). *Run length* is one of several different measures that could be used to assess significance. We conducted these analyses on three other measures that provide converging evidence; the results of these additional analyses are available from the authors.

### Discussion

These data demonstrate clearly that by 8 months infants can store information in VSTM using a task that closely resembles the adult one-shot change detection task. This task provides a purer measure of VSTM than previous tasks. In addition, we replicated the rapid developmental change in infants' VSTM for the identities of individual items in a multiple-item array. As has been found previously using the simultaneous streams task, 6-month-old failed to respond to changes of item identities in these multiple-item arrays whereas 8-month-old infants did respond to those changes.

Moreover, these results demonstrate that 8-month-old infants actually recognized which item changed, suggesting that they had individuated the items rather than storing a global representation of the entire array (Brady and Alvarez, 2011; Aly and Yonelinas, 2012). We are not proposing that 8-month-old infants in our task had a conscious awareness of what had changed, but rather that this information was able to guide their looking behavior.

Finally, the results reveal the time course of infants' responding in this task. The time course analyses rule out the possibility that the 6-month-old infants briefly looked toward the changed item and then looked away. They also rule out the possibility that 6-month-old infants simply took more time to detect the change than did the 8-month-old infants. That is, whereas 8-month-old infants exhibited a significant preference within 500 ms of the onset of the test array, 6-month-old infants did not exhibit a significant preference at any point within the first 1500 ms of the test array. It seems unlikely that a preference would have emerged even later, because (1) the overall amount of looking toward the two test items declined substantially after 1500 ms, (2) the longer the test array is on the screen, the less likely it is that an observer would still have the sample items available in VSTM, and (3) visual inspection of the time course for the 6-month-old infants over the entire 3000 ms test period did not reveal an increase in systematic preference for the changed item after the analysis window.

## Experiment 2

Although 6-month-old infants did not exhibit a significant change preference in Experiment 1, this cannot be used as evidence that they do not have a functional VSTM system at all. That is, they may have a functional VSTM system but be unable to use it under the conditions of Experiment 1. Moreover, a complete lack of VSTM in these infants seems unlikely given evidence of short-term memory for color and other features at 6 months in other tasks (Gilmore and Johnson, 1995; Rose et al., 2001) and evidence of VSTM from the simultaneous streams task at 6 months with single-item arrays (Ross-Sheehy et al., 2003).

As discussed in the Introduction, we instead propose that 6-month-old infants can store information in VSTM but have difficulty rapidly individuating multiple items in the sample array. To test this hypothesis, Experiment 2 was designed to determine whether 6-month-old infants would exhibit evidence of VSTM in our one-shot task when no individuation was necessary to encode the sample array. Specifically, Experiment 2 replicated the paradigm of Experiment 1 with one change: the sample array consisted of two identically colored squares instead of two differently colored squares, eliminating the need to individuate the two squares. The test array then consisted of one item of this same color and one item of a new color. If 6-month-old infants can store the color of the two identical sample items in VSTM—even without individuating the two sample items—they should be able to detect the new color value the test array.

Note, however, that although infants need not individuate items during the sample, they must be able to individuate the items in the *test* array to exhibit a preference for the new color. That is, to show a preference in the test array, infants must perceive the two different test colors, localize the new color, and make a selective eye movement to that color. However, this may not be as challenging as individuating the representations in VSTM. Thus, although we predicted that 6-month old infants would show a preference for the changed item in Experiment 2, we also expected that they would show some evidence of difficulty in individuating items in the test array (e.g., a slowing in the onset of the change preference relative to the 8-month-old infants tested in Experiment 1).

### Method

#### Participants

Twenty-four 6-month-old infants participated (*M* = 187.46 days, *SD* = 6.04, 13 boys), recruited as in Experiment 1. Fifteen infants were White, 1 infant was Asian, 3 infants were mixed race, and 5 infants did not have race reported. Seven infants were Hispanic. All mothers had graduated high school, and 16 mothers had earned at least a Bachelors degree. Five additional infants were tested but not included in the final analyses because of a failure to contribute sufficient numbers of trials (*n* = 2), failure to calibrate (*n* = 1), having a strong side bias (93% of all looking was to one side, *n* = 1), or change preference score more than 2.5 SD from the mean (*n* = 1).

#### Apparatus, stimuli, and procedure

All aspects of the apparatus, stimulus, and procedure were identical to those of Experiment 1, except that in the initial sample array the two squares were the same color. One of the two squares in the test array matched this color and the other was a different color.

### Results

We used the same inclusion criteria as in Experiment 1 and the same method to identify an analysis window (between 200 and 1717 ms after the onset of the test array see Figure 2B; we obtained the identical results when we used the window from Experiment 1). We first compared basic looking behavior between Experiments 1 and 2, irrespective of change preferences, to ensure that there were no large differences that might contaminate the change preference analyses. These comparisons revealed no differences in the number of trials completed (Experiment 2, *M* = 28.63, *SD* = 15.94), duration of looking during the sample [Experiment 2, 750 ms, *SD* = 67.39, Experiment 1, *M* = 717.31 ms, *SD* = 83.16, *t*_(48)_ = 1.52, *p* = 0.14, *d* = 0.43], or duration of looking during the analysis window of the test period, [Experiment 2, *M* = 1021.88 ms, *SD* = 323.82, Experiment 1, *M* = 884.62 ms, *SD* = 220.54, *t*_(48)_ = 1.76, *p* = 0.08, *d* = 0.50; when using the Experiment 1 analysis window, duration of looking in Experiment 2 was 929.86 ms, *SD* = 281.53]. Thus, 6-month-old infants did not differ in their general attention to these displays in the two experiments.

The average change preference score for infants in Experiment 2 during the analysis window for the test period is in Figure 3. These infants had a change preference score that was significantly greater than chance, *t*_(23)_ = 2.31, *p* = 0.03, *d* = 0.47.

We conducted the same time-course analysis described for Experiment 1. At the beginning of the pre-change period, these infants had one sample in which they significantly preferred the item that would not change. This was not a systematic preference, and our permutation analyses did not yield significant runs of preference during this period. Therefore, when the changed occurred, these infants did not prefer one side to the other.

During the post-change period, these infants exhibited a reliable preference for the changed item. The change preference scores at each sample point are shown in Figure 4B. A substantial preference for the changed item was observed at many points following the test array, with 95% confidence intervals that did not include chance and many significant *t*-values; uncorrected *t*-tests confirmed that the preference was significantly greater than chance for many samples.

The null distribution for the maximum run length is presented in Figure 5B; to be considered significant, a run of individually significant preference scores must be at least 16 samples long (just as was found for the 6-month-old infants in Experiment 1). The infant data revealed a run of 32 significant samples from 516.67 to 1050 ms after the onset of the test array (see Figure 4B). Thus, 6-month-old infants exhibited a sustained period of looking toward the changed item in the test array that exceeded the duration expected by chance. However, this period began later and ended earlier than the period of significant change preference found for 8-month-old infants in Experiment 1.

### Discussion

Experiment 2 replicated previous studies (Ross-Sheehy et al., 2003; Oakes et al., 2006), showing that 6-month-old infants can encode and store color in VSTM under some conditions. This shows that the present one-shot paradigm is a sensitive means of measuring VSTM in 6-month-old infants. The failure of the 6-month-old infants in Experiment 1 was not, therefore, the result of a general inability to demonstrate a novelty preference in this task (e.g., owing to poor oculomotor control). Instead, their failure was a result of the fact that two different colors were presented in the sample array and had to be individuated in VSTM. When the need to individuate two colors was eliminated in Experiment 2, infants were able to demonstrate a preference by consistently shifting gaze toward the changed item.

Interestingly, infants' response to the change in the multiple-item test array was slower and less robust than was the response of 8-month-old infants in Experiment 1, despite the fact that the encoding portion of the task was simplified. This may indicate that the 6-month-old infants also had some difficulty individuating the two colored squares in the test array.

## General discussion

We had two goals in this study. First, we sought to develop a purer assessment of VSTM in infancy. We present here a new *one-shot change detection* task that more closely resembles the task used to study VSTM in adults, along with a new approach for quantifying the time course of the change-related eye movements. Second, using this task, we sought to further understand of the rapid change in VSTM that occurs between 6 and 8 months. We will discuss each of these in the following sections.

First, we introduced and demonstrated the utility of a new method for the study of VSTM in infants. Our *one-shot change detection task* is a significant advance because the timing is similar to that in the change detection tasks used with adults, and it simulates the brief exposures and short delays that occur in natural vision, in which brief periods of fixation are separated by saccades and blinks. Thus, this is the first study to definitively show that infants can store information in VSTM, defined as a system that can rapidly form representations of a small number of items and maintain these representations across disruptions of the sensory input (see Luck, 2008). These properties of VSTM are very important if this memory system is to be used in visually guided behavior. Because gaze typically shifts every 200–500 ms during natural scene viewing (at least in adults, Henderson, 2008), the use of VSTM in visually guided behavior requires that the representations must be created rapidly, must be able to persist across short delays, and must be compared with a spatially shifted post-saccade visual input. We observed VSTM in both 6-month-old infants (in Experiment 2) and 8-month-old infants (Experiment 1).

Moreover, using this task, we replicated the rapid developmental change in VSTM between 6 and 8 months previously observed using the *simultaneous streams change detection task* (Oakes et al., 2006, 2009), thus confirming the role of VSTM in that task. These observed developmental changes also converge with results obtained in very different experimental paradigms, suggesting an important and general developmental transition in infants' memory of visual stimuli in the first postnatal year. For example, using a variation of a novelty preference task in which the study period was extremely short, Rose et al. (2001) observed capacity changes in infants' short-term memory between 7 and 12 months of age. Similarly, a study using a 3-well hiding task found significant changes in infants' ability to retain the hiding location between 7 and 9 months of age (Reznick et al., 1998). Finally, in a WM task in which infants had several seconds to encode the location of an object, Káldy and Leslie (2003, 2005) found evidence of developmental changes in binding in WM between 6 and 9 months of age. The consistency in the developmental trajectory across studies is remarkable considering differences in the measures and in the durations of the encoding period and retention interval. It is possible that the common factor in these findings is the developmental change in VSTM, although it remains to be seen whether the common developmental trajectory observed across these studies reflects the development of the same underlying mechanism.

The analytic procedures we introduce here also provide new understanding into VSTM in infancy. Unlike previous procedures in which conclusions are drawn based on infants' responding pooled over long periods of looking, here we examined infants' moment-by-moment looking during periods of a few seconds, adapting permutation analyses from the ERP literature (Groppe et al., 2011; Maris, 2012). These analyses confirmed the results of analyses of the summary of infants' behavior across the trial. By examining infants' performance on each data sample we gained additional information about their performance. We confirmed that at no point did 6-month-old infants in Experiment 1 prefer the changed item, demonstrating that their failure to show a significant preference was not an artifact of the way we averaged or summarized the data. We also showed that infants' preference was characterized by a prolonged period of preference. And, we observed that the preference emerged earlier for 8-month-old infants in Experiment 1 than it did for the 6-month-old infants in Experiment 2. Clearly, this procedure and analytic approach can provide a richer understanding of infants' change preference.

With respect to our second goal, this investigation contributes to our understanding of what is developing in VSTM between 6 and 8 months. In Experiment 2, we found at 6 months of age infants can store identity information in VSTM, consistent with findings from other studies using other procedures that reveal the emergence of related memory abilities by 6 months (Gilmore and Johnson, 1995; Reznick et al., 1998, 2004; Ross-Sheehy et al., 2003, 2011; Káldy and Leslie, 2005; Cheries et al., 2006). But, as in previous studies, we observed here that 6-month-old infants' change detection is restricted to cases in which the to-be-stored items are identical (Experiment 2); at this age, infants failed to exhibit VSTM when the two items in the sample were different colors (Experiment 1). This finding converges with prior data from the simultaneous stream task in which 6-month-old infants have difficulty rapidly encoding and retaining the features of multiple individual items (Ross-Sheehy et al., 2003; Oakes et al., 2011), even when every item in the array changes color on each cycle (Oakes et al., 2006, 2009).

As in previous studies, we observed here that infants older than 6 months of age can detect a change in these multiple-item arrays. Studies using the simultaneous streams paradigm have shown that between 7 and 8 months infants can detect changes in multiple-item arrays (Oakes et al., 2006, 2009), just as we observed in Experiment 1. Thus, we replicated the rapid developmental change in VSTM between 6 and 8 months. But, because change detection in our task is reflected in infants' preference *for the item that changed*, our results show that infants not only recognized *that* a change had occurred, but also that they identified *which item* had changed. In both experiments, infants responded by looking significantly longer toward the changed item than toward the unchanged item, indicating that they recognized and preferred the new color value during the test period. In the simultaneous stream task, detection of the change is measured by infants' preference for the *stream* that involves the change.

This is an important advance over the simultaneous streams task and provides a potential mechanism for the apparent change in VSTM capacity from 6 to 8 months. Specifically, the overall pattern of results suggests a developmental change in infants' ability to rapidly *individuate* multiple items during VSTM encoding between 6 and 8 months. At 6 months, infants can recognize change in multiple item arrays when that change does not require the rapid storage of individual items in those arrays. In the present investigation, 6-month-old infants responded to the change in Experiment 2 when the two items were initially the same color. Thus, infants could have encoded some global property of the array, rather than the features of each individual item. Similar success by 6-month-old infants has been observed when multiple item arrays were constructed of items all of the same color (e.g., 3 red items changed to 3 blue items which changed to 3 green items, and so on, Oakes et al., 2006) or when the change in individual items changed the overall configuration or shape of the array (Oakes et al., 2011). Thus, it appears that 6-month-old infants fail to detect a change in multiple item arrays when detecting that change requires that the to-be-encoded items be rapidly individuated. Consistent with this proposal, young infants do show evidence of VSTM for the color of a rotating object in an array of static colored objects (Ross-Sheehy et al., 2011), presumably because the rotating object attracts attention and can therefore be more easily individuated from the other objects. It is possible that attentional changes underlie these individuation processes, and that infants become better at individuating as they develop the ability to attend to items in multiple item arrays. Attention manipulations, such as that used by Ross-Sheehy et al. (2011) may increase young infants' sensitivity to changes in the items in this one-shot task. This should be addressed in future research.

This object individuation process is thought to be critically important in processing objects in natural visual displays, which typically contain multiple overlapping objects. Individuation may be necessary before items can be encoded into VSTM (Xu and Chun, 2009) and may underlie the capacity limitations in a number of tasks including VSTM and subitizing (Melcher and Piazza, 2011; Ester et al., 2012). Moreover, the posterior parietal cortex is important for these individuation processes in adults (Xu, 2009; Xu and Chun, 2009), and the extant evidence suggests dramatic changes in parietal regions in human infants in the first 6 months of postnatal development. For example, there are increases in glucose metabolism in this region during the second and third postnatal month (Chugani, 1999) and there is evidence of myelination of this region between 4 and 6 months (Deoni et al., 2011). Therefore, the regions of the brain thought to be central to individuation processes develop during the same time period as we observe changes in infants' ability to represent in VSTM the identity of individuated items. Of course, VSTM does not reach adult levels at the end of infancy, but continues to increase across childhood (Simmering, 2012). Understanding the source of these longer term developmental changes is beyond the scope of the present discussion, but it is likely that additional developmental changes in the brain mechanisms responsible for attention and individuation processes continue to contribute to changes in children's VSTM abilities.

It is important to point out that the present findings seem to contrast with a study reported by Blaser and Káldy (2010) using a “partial report” measure to assess the capacity of infants' iconic memory. The method used in that study was similar in many ways to our one-shot task: infants first were presented with a brief display of multiple items, all of different colors; two of the items disappeared for a brief retention interval; and when the two items reappeared, one had changed color. Six-month-old infants in this procedure preferred the changed item to the unchanged item when there were as many as 6 items on the screen, leading Blaser and Káldy to conclude that 6-month-old infants' iconic memory has a capacity of 5 items. The procedure used in these studies also differed in important ways. Blaser and Káldy's goal was to assess *iconic* memory; they used a procedure adapted from an adult procedure in which the sudden offset of the two items cued the infant to encode those items in VSTM; because the items were not present during the encoding, evidence that infants remembered them suggested they had been stored in iconic memory. Thus, to succeed in this task, they must form an iconic memory of 5 items, but they need only store 2 items in VSTM. Our study, in contrast, was designed to be like the change detection task used in adult research on VSTM (Luck and Vogel, 1997); as in the adult work, infants are provided an opportunity to encode in VSTM items that are briefly available on the display. The point is that although Blaser and Káldy (2010) appear to have obtained evidence that 6-month-old infants successfully encoded two items in VSTM (two items disappeared, and when the items reappeared infants looked longer at the changed item), there are significant differences in the procedures that make it difficult to directly compare the results. In addition to the fact that we used a change detection procedure and Blaser and Káldy used a partial report procedure, the relative size of the to-be-remembered items were considerably different—11° by 11° in our study and 3° by 3° in Blaser and Káldy's study—as were the distance between the two items on the screen. Such differences may have important consequences for how infants attend to, perceive, and individuate the items in the displays. For example, 6-month-old infants may be unable to group our relatively large and distant objects, but may more easily group or unify the smaller and closer objects used by Blaser and Káldy. As a result, Blaser and Káldy may have measured infants' preference for the changed *part* of a single multi-part item, and we measured infants' preference for the changed identity of one item in a multi-item array. Indeed, we previously found that 6-month-old infants stored three locations in VSTM if these locations form a distinct object (i.e., a triangle) but not if they must be stored as separate, individuated locations (Oakes et al., 2011). Future research is needed to assess the validity of this explanation.

These results provide new evidence about the development of VSTM for multiple objects in infancy. They suggest that developmental differences in infants' responses to changes in multiple-item arrays reflect differences in the ability to individuate items in these arrays. Item individuation is thought to be a key process in the perception of natural visual scenes and may also play a significant role in determining the capacity of VSTM in adults. The present results not only provide insight into the fact that infants' VSTM develops during the first year, but begin to address questions of how this important ability develops.

### Conflict of interest statement

The authors declare that the research was conducted in the absence of any commercial or financial relationships that could be construed as a potential conflict of interest.
